# Anatomical characteristics of the styloid process in cerebral infarction related to carotid artery dissection: a case-control study

**DOI:** 10.3389/fneur.2025.1573667

**Published:** 2025-06-17

**Authors:** Shuna Shi, Limei Wang, Haiyang Luo, Zhenling Fu, Yutao Liu

**Affiliations:** Department of Neurology, The First Affiliated Hospital of Zhengzhou University, Zhengzhou University, Zhengzhou, Henan, China

**Keywords:** styloid process length, styloid process inclination angle, styloid process syndrome, carotid dissection, internal carotid

## Abstract

**Purpose:**

To verify the hypothesis that length and inclination angle of the styloid process may lead to cerebral infarction related to carotid artery dissection through comparison of the length and inclination angle of the styloid process in middle-aged and young stroke patients undergoing carotid artery dissection, vertebral artery dissection, and non-dissection controls.

**Methods:**

This was a Retrospective, single-center, case-control study that enrolled patients with cerebral infarction related to internal carotid artery dissection, vertebral artery dissection, and non-dissection controls. Eighteen patients with carotid artery dissection patients (cases) were compared with 34 sex-matched patients with vertebral artery dissection (G1) and 55 sex-matched patients without dissection (G2). The length and inclination angle of the styloid process were measured using Computed tomography angiography images. Differences between groups were estimated using the Student's *t-*test.

**Results:**

Styloid process length ipsilateral to the carotid artery dissection was not significantly longer than that of the contralateral side of the cases (*p* > 0.05), or to those of the ipsilateral and contralateral sides in patients with vertebral artery dissection (*p* > 0.05). The styloid process length ipsilateral to the dissection was significantly longer than those of the left and right sides of control patients (*p* < 0.05 for both), while the inclination angle of the cases was significantly larger than those of the ipsilateral and contralateral sides of the vertebral artery dissection patients (*p* < 0.05), and to the left and right sides of control patients (both *p* < 0.05).

**Conclusion:**

Long styloid process length and a large inclination angle appear to be risk factors for carotid artery dissection (CAD).

## 1 Introduction

Carotid artery dissection (CAD) is a common cause of stroke in young individuals, attributable to both genetic and environmental factors. Environmental factors include neck activities, such as sudden braking, neck massage, yoga, coughing, sneezing, vomiting, and wall brushing. Other related factors include infection, hypertension, hypercholesterolemia, and obesity. Furthermore, styloid process syndrome (SPS) is associated with CAD, and the styloid process (SP) is gradually receiving attention. Most cases of SPS are caused by sore throat, foreign body sensation in the throat, and pain during neck turning; as such, patients seeking treatment at the head and neck surgery clinic may overlook symptoms such as internal CAD or even secondary acute cerebral infarction; however, related research is lacking. This study further explored the pathogenesis of CAD by analyzing the relationship between SP length, inclination angle, and internal CAD -induced cerebral infarction.

SPS most often occurs in women aged 30–50. The cause may be ossification of the styloid hyoid ligament caused by abnormal levels of calcium, phosphorus, vitamin D, or parathyroid hormone (1), SPS may also be congenital or related to familial genetic factors (2).

SPS is divided into the classic and SP arterial type (3); the former of which commonly occurs after tonsillectomy, causing pain in the ipsilateral face, head, and neck; foreign body sensation in the throat; tinnitus; swallowing disorders, etc. (4). Imaging manifestations of the arterial-type SP include internal CAD, pseudoaneurysm, carotid artery stenosis or the carotid stent broke (5, 6).

The structure of the SP around the carotid artery requires attention, particularly the length and inclination angle of the styloid process (7). The extended SP compresses the internal carotid artery, leading to dissection. Studies have further suggested that when the SP length exceeds 31.15 mm, the risk of CAD increases fourfold (8). Several cases of ischemic stroke secondary to SPS with bilateral CAD have also been reported (9–11). SPS is not only related to an elongated styloid process, but also to abnormal angles of the SP or variations in the vascular anatomy of the carotid artery itself, which can cause compression of the internal carotid artery by the bone process, leading to CAD (12). Some studies suggest that the proximity of the styloid process to the carotid artery and the mechanical action of the styloid process on the ligaments and muscles surrounding the ipsilateral internal carotid artery are related to the occurrence of carotid artery dissection (1, 3, 13).

The manifestations of CAD-related cerebral infarction in SPS may involve multiple mechanisms, including (1) cerebral tissue ischemia directly caused by SP compression of the internal carotid artery; (2) damage to the inner wall of the carotid artery, resulting in carotid embolism or internal CAD; and (3) vasovagal nerve response. Research has further shown that an extended SP enhances the lateral support of the stylopharyngeal muscle, causing more pronounced compression of the internal carotid artery during neck rotation (5). Consequently, patients with symptoms of CAD, such as head and neck headache, Horner's syndrome, limb hemiplegia, hemiparesis, aphasia, and consciousness disorders, need to be differentiated from those with SPS.

Computed tomography angiography (CTA) and 3D reconstruction are widely recognized as the best methods for diagnosing SPS (14), as they can visually observe the length, inclination angle, and relationship of the SP with the internal carotid artery. However, one meta-analysis indicated that imaging examination methods do not affect the diagnosis of SPS (15). The research objective was to investigate the correlation between the length and angle of the styloid process and carotid artery dissection related cerebral infarction.

## 2 Methods

### 2.1 Study participants

Cases and controls were selected from patients, aged 18–55 years, consecutively admitted to the stroke unit of our hospital, from January 1, 2022, to January 1,2024. CAD and VAD diagnosis was verified by two specialists, They observed endometrial patches, intramural hematoma, double lumen sign, and rat tail sign through CTA or high-resolution MRI (16–18). Ethical approval was obtained from the relevant institutional review board (IRB:2021-KY-1289-001); however, the need for informed consent was waived due to the retrospective study design.

The inclusion criteria were as follows: ① Patients with acute cerebral infarction; ② Age between 18 and 55 years old; ③ underwent completed head magnetic resonance imaging (MRI), MR angiography (MRA), and head and neck combined with CTA in our hospital. Patients were divided into the CAD, VAD, and other cause groups, according to the TOAST classification. Patients with severe cardiovascular disease, neurological and psychiatric diseases, neuromuscular diseases, SP fractures, CAD in the form of pseudoaneurysms, or fibromuscular dysplasia were excluded.

#### 2.1.1 Groups

Enrolled patients were categorized into the observation group and two control groups, as follows: (1) observation group: patients with CAD, excluding those with internal carotid artery aneurysms, muscle fiber dysplasia, and vasculitis. (2) Control group 1 (G1): patients with vertebral artery dissection (VAD), excluding vertebral artery aneurysm. (3) Control group 2 (G2): age-and sex-matched patients with non-dissection cerebral infarction, excluding those with internal carotid artery aneurysms and those who underwent internal carotid artery intervention surgery.

### 2.2 Measurements of styloid process length and styloid process inclination angle

**Figure 1:**
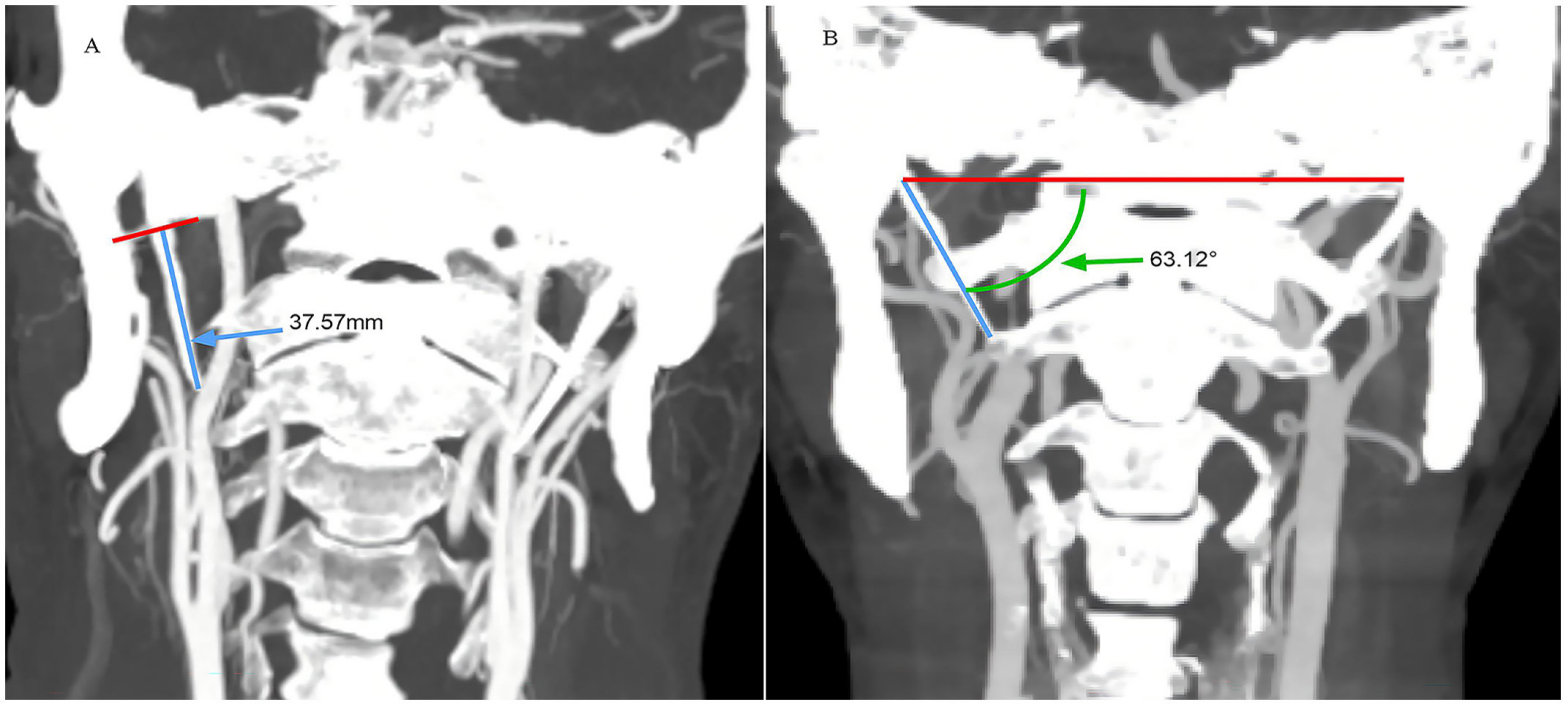
Examples of measurements of styloid process length [**(A)** small blue arrow] and styloid process inclination angle [**(B)** small green arrow].

CT imaging was performed using a Siemens 64 slice double helix machine, with patients in the supine position, with the lower jaw elevated and the orbital line perpendicular to the scanning bed. It is generally believed that a large inclination angle can easily compress the internal carotid artery; therefore, inclination angle measurement was performed. Measurements included bilateral styloid process length and angulation of the process relative to midline structures (SP inclination angle) (Figure 1). The angle formed by the line connecting the tip of the SP and the midpoint of the SP root to the horizontal line of the skull base was measured (Figure 1). Multiple studies have used this measurement method to analyze the relationship between styloid process length and internal angle and carotid artery dissection (8, 19). The measurement was performed by two neurology experts (Operator 1 and Operator 2) and the average value was taken.

### 2.3 Collect data

A researcher recorded the basic characteristics (age, BMI, CRP, glycated hemoglobin, or cholesterol levels, neck massage or stretching, sex, hypertension, diabetes, smoking) of cases based on medical records, and the length and angle of the styloid process were collected for cases by two independent researchers, taking the average. Neck massage or stretching defined: Cervical chiropractic neck manipulations (using hands or massagers to massage the neck), heavy lifting, sport associated injuries which are the most common etiologies (20, 21).

### 2.4 Statistics

All statistical analyses were performed using the Statistical Package for Social Sciences) IBM SPSS Statistics ver27. Quantitative data (age, BMI, CRP, glycated hemoglobin, or cholesterol levels, the SP length and SP inclination angle) were expressed as mean ± standard deviation (mean ± SD), and Student's *t*-test was used for statistical difference analysis. Qualitative datas such as sex, hypertension, diabetes, smoking, neck massage or stretching were expressed as percentage (%) and Chi square test or Fisher's exact test. Statistical significance was set at *p* < 0.05. Bland Altman analysis was used to measure the interrater agreement.

## 3 Results

Basic information about the CAD, VAD, and non-dissection groups was presented in Table 1. There were no statistically significant differences in age, sex, BMI, hypertension, diabetes, smoking, CRP, glycated hemoglobin, or cholesterol levels (*P* > 0.05). However, statistically significant differences were found in neck massage or stretching between the CAD and VAD groups (*P* = 0.03), and in moderate alcohol consumption (men: ≤ 3 units/day; women: ≤ 2 units/day) between the CAD and non-arterial dissection (*P* = 0.01). Compared to the VAD group, there were more cases of neck massage or stretching in the CAD group; and more cases of moderate alcohol consumption history in non-dissection groups, however, moderate alcohol consumption was not identified as a risk factor for an abnormal SP.

**Table 1:** Baseline characteristics of cases and controls.

**Clinical characteristics**	**CAD (*n =* 18) x ±SD or p**	**VAD (*n =* 34) x ±SD or p**	**G2 (*n =* 55) x ±SD or p**	***P*-value**
Age	44.44 ± 11.25	39.85 ± 13.13	46.47 ± 7.52	*P*1 = 0.21 *p*2 = 0.48
Sex *m* (%)	14/18	26/34	43/55	*P*1 = 0.92, *p*2 = 0.97
BMI	26.14 ± 3.7	25.31 ± 3.04	26.26 ± 3.56	*P*1 = 0.39, *p*2 = 0.90
Neck massage or stretching (%)	0/18	1/34	0/55	*P*1 = 0.03, *p*2 = 1.0
Hypertension (%)	6/18	10/34	29/55	*P*1 = 0.78, *p*2 = 0.15
Diabetes mellitus (%)	0/18	5/34	8/55	*P*1 = 0.13, *p*2 = 0.10
Past smoking (%)	3/18	11/34	20/55	*P*1 = 0.25, *p*2 = 0.12
Moderate alcohol consumption (%)	1/18	7/34	15/55	*P*1 = 0.08, *p*2 = 0.01
CRP	2.93 ± 2.97	4.02 ± 6.42	3.00 ± 4.72	*P*1 = 0.51, *p*2 = 0.96
Glycosylated hemoglobin	5.86 ± 1.06	5.88 ± 1.88	6.37 ± 1.30	*P*1 = 0.95, *p*2 = 0.14
Total cholesterol	4.00 ± 1.74	3.74 ± 1.02	3.89 ± 0.92	*P*1 = 0.51, *p*2 = 0.74

*p*1: CAD vs. VAD; *p*2: CAD vs. Control group 1. x, mean; *p*, proportion; BMI, body mass index; CRP, C-reactive protein.

**Figure 2:**
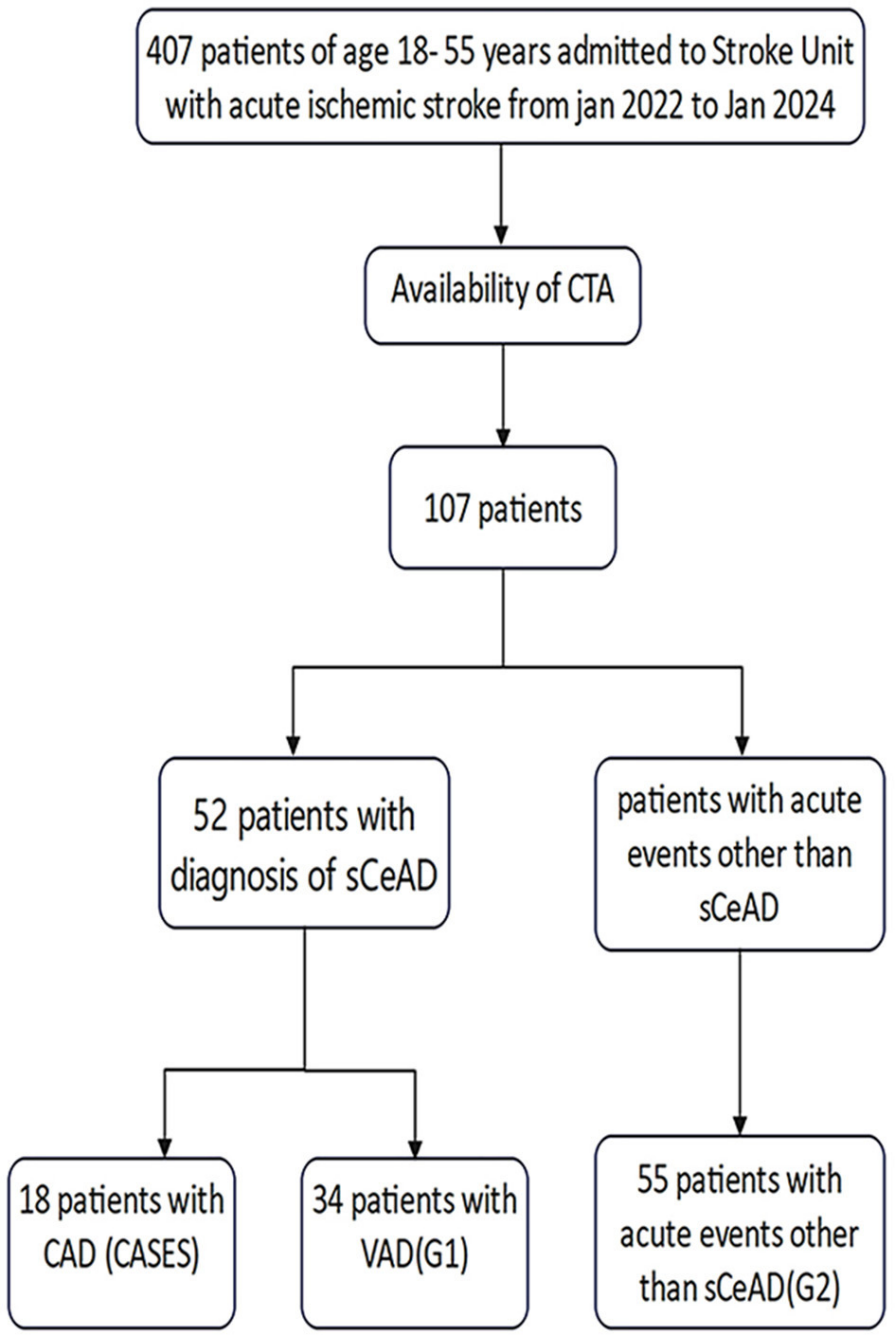
The flowchart of patient selection.

Among 407 cases of acute cerebral infarction aged 18–35, 107 cases completed CT-angiography (CTA) in our hospital and were selected for the study. We included and analyzed 18 patients with CAD,34 with VAD (age- and sex-matched with CAD patients, G1), and 55 non-dissection control group patients (age/sex-matched, G2, Figure 2). The CAD group comprised 14 of 18 males (78%) and 4 of 18 females (22%), with an average age of 44.44 years (±11.25). There were 26 males and 8 females in the VAD group, with an average age of 39.85 years (±13.13); There were 43 males and 12 females in the non- arterial dissection patient group, with an average age of 46.47 years (± 7.52). There are more males than females in the CAD and VAD groups.

**Figure 3:**
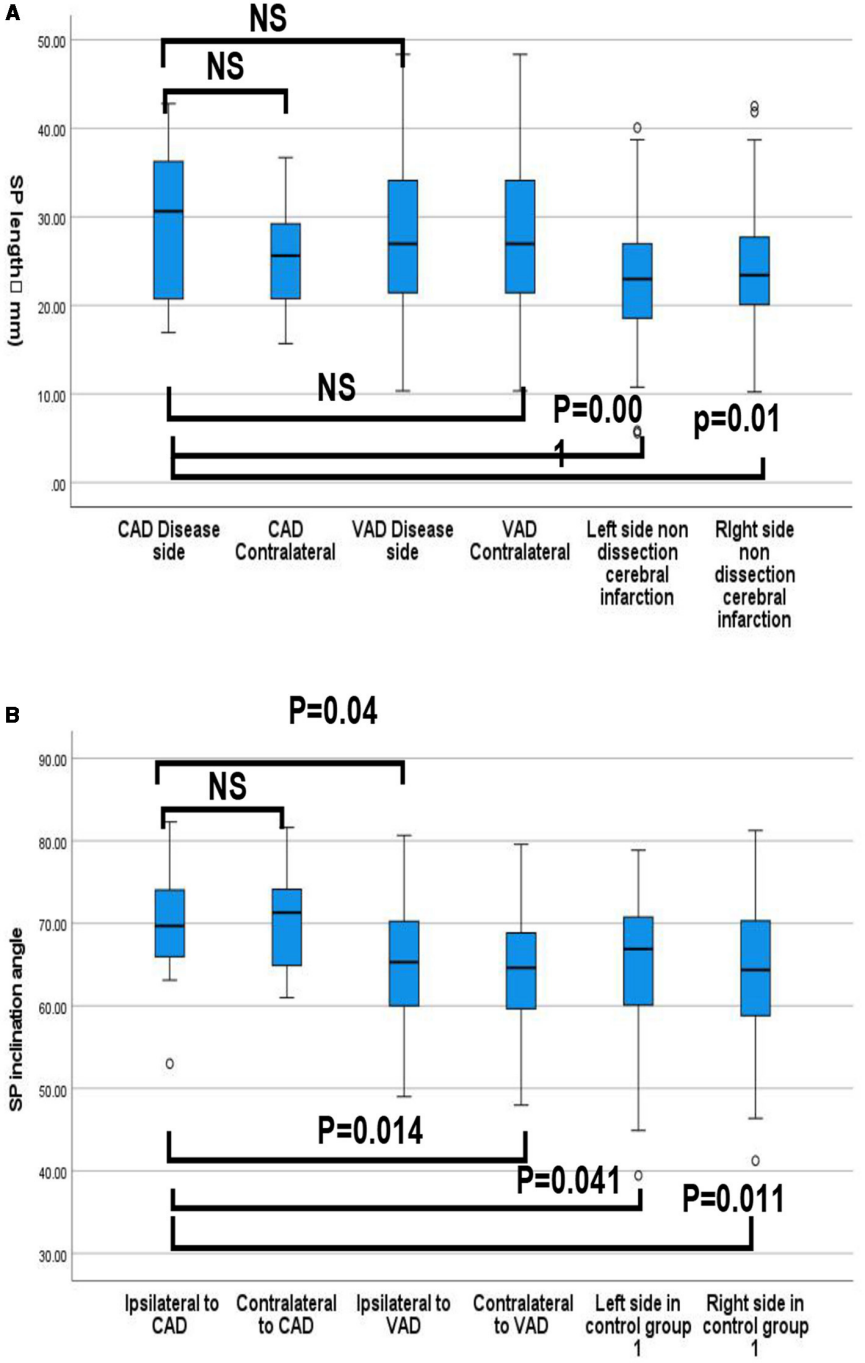
**(A)** Quartile distribution of styloid process length (mm) between cases and controls (CAD groups, VAD groups, Non-arterial dissection groups. **(B)** Quartile distribution of the styloid process Inclination angle (°) between cases and controls (CAD groups, VAD groups, Non-arterial dissection groups. *P*-values refer to independent *t-*student tests for comparison of means (ns: non-significant, *p* > 0.05).

The length of the SP in the CAD group was longer than that in the non-dissection group, with an ipsilateral SP length of 29.78 mm (± 8.19) and a contralateral SP length of 25.61 mm (± 6.31). There was no statistically significant difference in the length of the SP between the carotid dissection and contralateral sides (*P* = 0.98). The length of the ipsilateral SP in the VAD group was 26.16 mm (±7.56), while the length of the contralateral SP in the CAD group was 28.40 mm (±9.42). There was no statistically significant difference in the length of the SP between the VAD side and contralateral side (*P* = 0.29). The length of the left SP was 22.63 mm (± 7.20), and the length of the right SP was 24.40 mm (±7.00) in non-stratified stroke patients, with no statistically significant difference between the two sides (*P* = 0.20). There was no significant difference in the length of the SP between the CAD side and the VAD side (*P* = 0.12); however, there was a significant difference in the length of the right SP between patients with CAD and those without dissection cerebral infarction (*P* = 0.01), and in the length of the left SP between patients with CAD and those without dissection cerebral infarction (P = 0.001) (Figure 3).

SP inclination of the ipsilateral side was not significantly larger compared to that of the contralateral side in CAD cases (69.76° ± 6.89 vs. 70.26° ± 5.62, *p* = 0.813), but was significantly larger than those of the ipsilateral (65.33 ± 73.4, *P* = 0.04) and contralateral (67.55° ± 7.05, *P* = 0.014) sides in the VAD groups, and to the left (65.09° ± 8.64, *p* = 0.041) and right (63.86 ± 8.72, *P* = 0.011) sides of non-dissection stroke patients.

**Figure 4:**
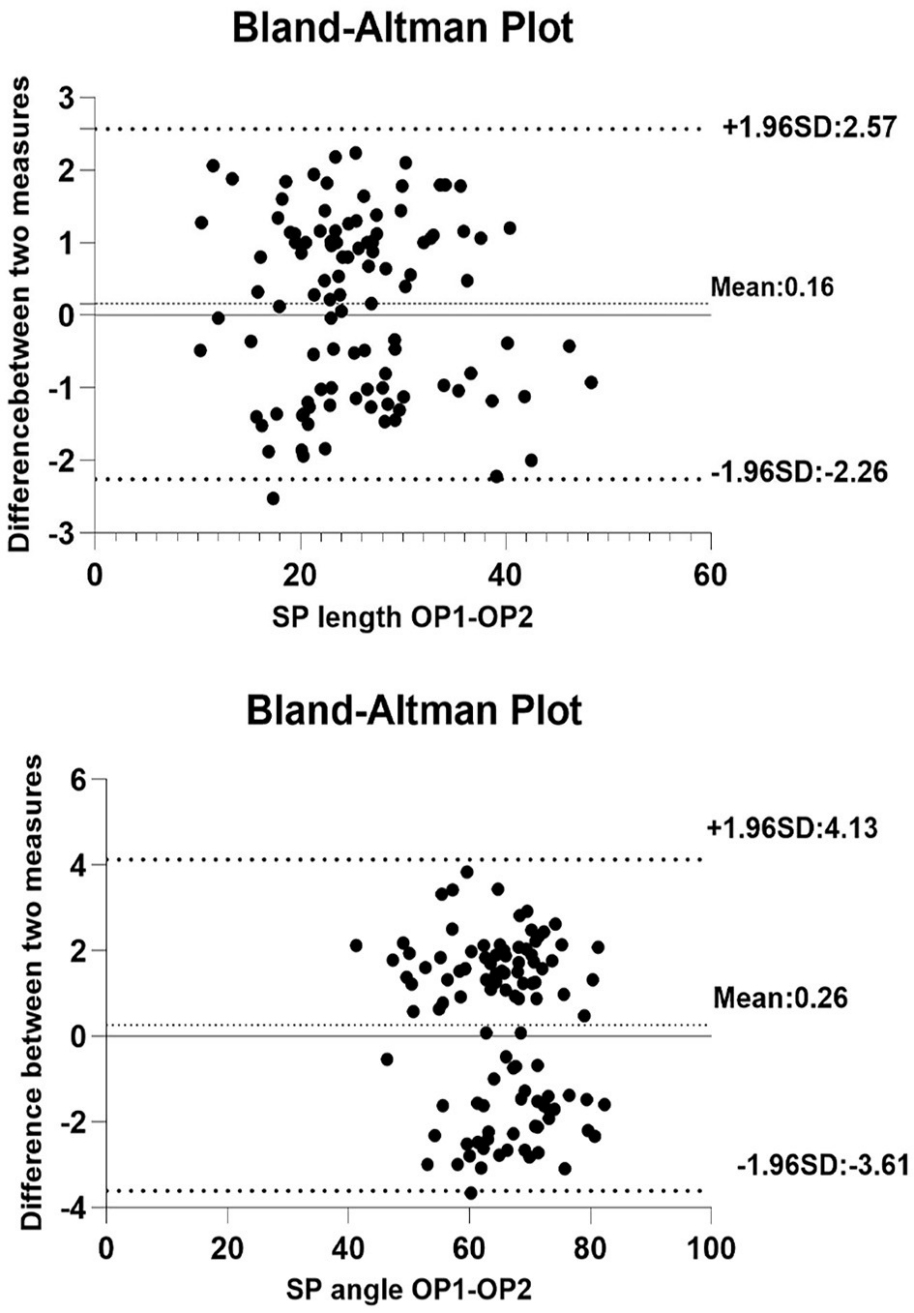
Bland Altman analysis: interrater agreement for styloid process length and angle (OP 1, operator 1; OP 2, operator 2).

Bland Altman analysis showed a good interrater agreement between the measurements independently taken by the two Neurologist: 95% of the differences between measurements are included within the limits of agreement (Figure 4).

## 4 Discussion

Our study confirmed data from prior studies suggesting that the SP length and inclination angles could be risk factors for CAD; furthermore, our results indicate that the SP length of patients with CAD is longer than that of non-dissection stroke patients, consistent with a previous study. In contrast to previous studies (8, 19, 22), we found that the characteristics of the SP contralateral to the ICAD were not significantly associated with dissection, which could explain the low frequency of bilateral ICAD. However, this association was only marginally significant, which could be related to the small sample size. Furthermore, unlike previous systematic reviews and meta-analyses (15), our research showed that there are significantly more male than female cases of CAD, which may be related to the single center design and small sample size of the present study. Further, our findings are targeted at young and middle-aged CAD patients, which does not fully represent the characteristics of Carotid artery dissection patients. The mechanical compression or friction between the SP and the carotid artery is caused by the anatomically long length and large inclination angle of the SP, combined with external factors, such as neck movement, coughing, and neck massage. This study found that the SP length and inclination angles may have potential mechanical effects in some CAD cases.

Consequently, the proposed measurement method is reliable. Measuring the length of the SP on head and neck CTA is more intuitive, and the inclination angle of the SP is not affected by the calcification of the hyoid bone SP. As a bony structure, the SP will not shift or change in the short term. We excluded patients with CAD in the form of pseudoaneurysms, as well as patients with muscle fiber dysplasia and SP fractures, in whom measurements are more difficult because of interference from multiple factors.

The distance between the SP and the carotid artery was not measured in this study because the anatomical variation of the SP is large, and the location could not be uniformly maintained. Moreover, the distance was small, and the results were easily affected by different measurements and the condition of the carotid hematoma.

The primary limitations of our study are as follows: Firstly, the single-center design could have introduced a patient selection bias. Some patients who visited our center for treatment had already completed head and neck CTA examinations outside the hospital or refused CTA examination; these patients were excluded to reduce measurement interference factors, resulting in a limited number of patients with CAD being screened out. Furthermore, 3D reconstruction CTA examination was not available because of limited conditions, and CTA examination was not performed at different angles of head rotation to directly demonstrate the mechanical effect of SP on the carotid artery. In addition, there may be a risk of carotid artery reinjury during neck rotation, and a meta-analysis showed that imaging examination methods do not affect the diagnosis of Styloid Process Syndrome (15).

Despite these limitations, this study opens up new insights, stimulates further explorations of the etiology and pathogenesis of dissection in young and middle-aged patients. Few long-term follow-up studies have been conducted on patients with CAD; therefore, reports on the recurrence of CAD are rare. Because of the many potential risk factors for CAD, including underlying connective tissue disease, infection, migraine, hyperhomoglutamatemia, genetic factors, and other environmental factors, the SP length and medial inclination angle may be risk factors. Based on the anatomical risk factors of the SP, when the head and neck rotation reaches a certain degree, adverse mechanical conditions will occur, leading to direct damage to the internal carotid artery by the styloid process. In follow-up studies, SP anatomy can be used as a follow-up factor.

## 5 Conclusions

Our research suggested that abnormal length and tilt angle of SP may be a risk factor for cerebral infarctions associated with CAD in young-and middle-aged patients. Consequently, the length and inclination angle of the SP should be considered when diagnosing and treating such patients. The anatomical relationship between the SP and the carotid artery before CAD stent surgery also needs to be considered to reduce or even avoid secondary damage to the carotid artery caused by SP after surgery. When asymptomatic elongation or an abnormal angle of the SP is observed, medication and surgical treatment are unnecessary. Before SP resection surgery, it is important to avoid inducing movements such as neck massage, sudden neck rotation, and neck stretching as much as possible.

## Data Availability

The original contributions presented in the study are included in the article/supplementary material, further inquiries can be directed to the corresponding author.
